# Subitizing and visual short-term memory in human and non-human species: a common shared system?

**DOI:** 10.3389/fpsyg.2012.00469

**Published:** 2012-11-02

**Authors:** Simone Cutini, Mario Bonato

**Affiliations:** Department of General Psychology, University of PadovaPadova, Italy

Numerical competence is widely spread across human and non-human species. Here we discuss the possibility that the similarities shown in the processing of small sets of items might be due to the characteristics of basic cognitive abilities for the processing of visual items, common to both human and non-human living beings.

Several species show a fast and accurate performance in judging the numerosity of small sets of items, an ability termed “subitizing” (Tomonaga and Matsuzawa, 2002; Agrillo et al., 2012). Regardless of whether one or two systems for the processing of non-symbolic magnitudes exist, the similar performances often observed across such diverse species have led to the hypothesis that there may be shared core systems supporting numerical abilities of non-human species and non-verbal numerical abilities of humans (Beran et al., 2011). A challenging question is whether these similarities in non-symbolic numerical information processing are due to numerical competences or if they are caused (in both human and non-human species) by the limits of the systems devoted to the processing of visual sets of items (visual short-term memory, VSTM). VSTM can retain a limited amount of information at one time and, in humans, it is typically investigated by means of change detection paradigms. In contrast, most of the paradigms investigating the neural mechanisms of VSTM in animals required the retention of just a single memorandum and, until recently, no study provided a direct comparison between humans and primates of VSTM capacity in a change detection task. This issue was addressed by Elmore et al. (2011) by testing humans and rhesus monkeys with the same change detection paradigm. A memory array (composed by colored circles or clip arts figures), was presented and, after a retention interval, a test array with two items appeared; the response consisted in choosing the “changed” item. Task difficulty was manipulated by varying the number of items presented in the memory array (i.e., the VSTM load). The authors were able to show that animals can perform a change-detection task with the same procedures/stimuli used for humans, and also succeeded in highlighting the qualitative similarities between the performance of monkeys and humans. This comparative study, as the one by Beran et al. (2011), is an interesting example suggesting that the use of identical procedures for humans and animals might provide fruitful insights on humans' cognitive performance: indeed, it strongly suggests the possibility to enlarge the research field of numerical cognition with truly multidisciplinary approaches, where conclusions made for one (non-human) species can be informative also for a second one (e.g., human).

To address the point we want to raise here, namely the fact that subitizing range cannot be considered as independent from VSTM capacity limits, the bridge across species needs to be complemented by a further bridge across cognitive abilities. The latter bridge has been provided by an influential behavioral experiment on humans (Piazza et al., 2011) which addressed the relation between the processing of non-symbolic magnitudes and VSTM. Beginning with the naïve observation that in humans the behavioral limits of subitizing and VSTM are strikingly similar (around 3–4 items), the authors adopted a sophisticated dual-task paradigm to determine whether these similar capacity limits are subserved by the same cognitive mechanisms and recruit the same resources. In each trial, participants performed two tasks: a counting task and a change detection VSTM task. Participants were first presented with a memory array of either two or four colored circles (low vs. high VSTM load), briefly replaced by a counting set ranging from one to eight items. This set was then masked and the participants were asked to report its numerosity (primary task). Finally, they were presented with a test array (same number of colored circles of the memory set) and performed a same–different judgment with respect to the memory set (secondary task). The amount of VSTM load selectively impaired performance in the counting task, by reducing the individual subitizing range, but had no effect on the estimation of large quantities; furthermore, the interference between the two tasks exhibited a predictable pattern, in line with the idea of a common capacity limit. This result suggests the presence of a domain general mechanism (i.e., multiple object individuation) shared by subitizing and VSTM; by recruiting the core resources that are characterized by a limited capacity, even an apparently basic ability like subitizing can be significantly impaired. From a broader perspective there is one main question that can be intuitively addressed: what is the comparison of non-symbolic magnitudes, if not the active maintenance of information conveyed by stimuli which are no longer in view?

Interestingly, the integrated view provided by the two investigations presented here sheds light on the advantages that a comparative study on VTSM and numerical cognition might grant both across and within species. Importantly, VSTM provides an essential link between perception and higher cognitive functions, and the role played by capacity limits of VSTM should not be neglected when investigating the basic numerical abilities both in humans and animals. In conclusion, given the mounting evidence that non-symbolic numerical processing and VSTM are intimately related, and given their qualitative similarities in humans and non-humans, we can argue that a comparative investigation on the relation between non-symbolic numerical processing and VSTM might provide dramatic advances in the understanding of the bases of numerical processing. This type of approach can open to the broader idea of an assessment of cognitive abilities and capacities in non-human species. It could also open to a fine-grained analysis of the ability to flexibly deploy attentional resources/capacity limits in such cognitive abilities. Finally, this approach might help to solve outstanding questions such as to discover whether subitizing and large numerosity estimation are related to two different mechanisms or whether they are only the two extremes of the same continuum, and whether non-symbolic numerical abilities and VTSM can be dissociated in animals.

## References

[B1] Agrillo C. Piffer L. Bisazza A. Butterworth B. (2012). Evidence for two numerical systems that are similar in humans and guppies. PLoS ONE 7:e31923 10.1371/journal.pone.0031923 22355405 PMC3280231

[B2] Beran M. J. Decker S. Schwartz A. Schultz N. (2011). Monkeys (*Macaca mulatta* and *Cebus apella*) and human adults and children (*Homo sapiens*) enumerate and compare subsets of moving stimuli based on numerosity. Front. Comp. Psychol. 2:61 10.3389/fpsyg.2011.00061 21716575 PMC3110735

[B3] Elmore L. C. Ma W. J. Magnotti J. F. Leising K. J. Passaro A. D. Katz J. S. (2011). Visual short-term memory compared in rhesus monkeys and humans. Curr. Biol. 21, 975–979 10.1016/j.cub.2011.04.031 21596568 PMC4634532

[B4] Piazza M. Fumarola A. Chinello A. Melcher D. (2011). Subitizing reflects visuo-spatial object individuation capacity. Cognition 121, 147–153 10.1016/j.cognition.2011.05.007 21679934

[B5] Tomonaga M. Matsuzawa T. (2002). Enumeration of briefly presented items by the chimpanzee (*Pan troglodytes*) and humans (*Homo sapiens*). Anim. Learn. Behav. 30, 143–157 12141135 10.3758/bf03192916

